# Deep Learning for Heart Sound Abnormality of Infants: Proof-of-Concept Study of 1D and 2D Representations †

**DOI:** 10.3390/children12091221

**Published:** 2025-09-12

**Authors:** Eashita Wazed, Jimin Lee, Hieyong Jeong

**Affiliations:** 1 Department of Artificial Intelligence Convergence, Chonnam National University, Gwangju 61186, Republic of Korea; eashitawazed@jnu.ac.kr; 2 SW Convergence Education Institute, Chosun University, Gwangju 61452, Republic of Korea; histhing@naver.com

**Keywords:** audio data processing, audio feature extraction, deep learning in cardiology, heart sound, heart function in infant, stethoscope

## Abstract

**Highlights:**

**What are the main findings?**

- This study proposes a novel multimodal deep learning framework that uniquely integrates both 1D time-series and 2D image representations of infant heart sounds, achieving a state-of-the-art accuracy of 98.91% for abnormality detection.
- The study systematically demonstrates that this fusion model not only improves classification performance over using either data representation alone but also significantly reduces the computational training cost compared to purely image-based models.

**What are the implications of the main findings?**

- This work’s primary implication is the potential for a more accessible and cost-effective method for early screening of congenital heart defects in infants, as the model’s high accuracy is achieved using audio from stethoscopes, which are more readily available than specialized equipment like ECGs.
- Academically, the research provides a significant insight for the field by demonstrating that a multimodal deep learning approach can surpass the performance of single-modality models while also being more computationally efficient, offering a promising direction for the development of future diagnostic AI.

**Abstract:**

**Introduction:** Advanced identification and intervention for Congenital Heart Defects (CHDs) in pediatric populations are crucial, as approximately 1% of neonates worldwide present with these conditions. Traditional methods of diagnosing CHDs often rely on stethoscope auscultation, which heavily depends on the clinician’s expertise and may lead to the oversight of subtle acoustic indicators. **Objectives:** This study introduces an innovative deep-learning framework designed for the early diagnosis of congenital heart disease. It utilizes time-series data obtained from cardiac auditory signals captured through stethoscopes. **Methods:** The audio signals were processed into time–frequency representations using Mel-Frequency Cepstral Coefficients (MFCCs). The architecture of the model combines Convolutional Neural Networks (CNNs) for effective feature extraction with Long Short-Term Memory (LSTM) networks to accurately model temporal dependencies. Impressively, the model achieved an accuracy of 98.91% in the early detection of CHDs. **Results:** While traditional diagnostic tools like Electrocardiograms (ECG) and Phonocardiograms (PCG) remain indispensable for confirming diagnoses, many AI studies have primarily targeted ECG and PCG datasets. This approach emphasizes the potential of cardiac acoustics for the early diagnosis of CHDs, which could lead to improved clinical outcomes for infants. Notably, the dataset used in this research is publicly available, enabling wider application and model training within the research community.

## 1. Introduction

Research and clinical findings indicate that heart disease can be inherited through genetic predispositions from parents [1]. Various genetic disorders may contribute to the transmission of heart disease across generations within a familial lineage. Early heart disease detection and diagnosis are critical for improving patient outcomes and longevity [2]. It is not uncommon for pediatric patients to inherit cardiac conditions without parental knowledge, and due to their limited communication abilities, these issues often remain undiagnosed until adulthood.

The standard modalities for diagnosing heart disease typically include Electrocardiograms (ECGs) and echocardiograms (Echo), which can be resource-intensive in terms of time and cost. For this reason, ECG and Echo are widely used for diagnosing heart disease [3]. However, these examination methods are only available in specialized hospitals, in addition to being costly, and in many cases, ECG detects heart abnormalities only after the disease has significantly progressed. In contrast, heart murmurs, stenosis, and valvular insufficiencies can be easily detected through heart sounds. In recent years, substantial research has focused on analyzing heart sounds, particularly utilizing Phonocardiogram (PCG) signals [4].

Studies have explored various machine learning architectures, such as Convolutional Neural Networks (CNNs) and Long Short-Term Memory (LSTM) networks. Additionally, methodologies involving Fast Fourier Transform (FFT) and Mel-Frequency Cepstral Coefficients (MFCCs) have been employed for feature extraction in these models. Future directions in this research area will focus on augmenting existing datasets, integrating a wider array of features, and referencing key works [1,2,3,4,5]. Some studies have leveraged the Wavelet Transform (DWT) [2] as a practical feature extraction technique and discussed the Short-Term Fourier Transform (STFT) [6] for signal analysis.

While numerous concise reviews on the application of deep learning models to ECG and PCG signals exist [7,8,9,10,11], there remains a notable gap concerning the analysis of heart sounds concerning audio data. The delayed diagnosis of myocardial infarctions often results in increased mortality rates due to the lack of timely medical intervention. This lag in treatment is frequently attributed to the high costs and labor-intensive nature of conventional ECG and Echo testing. Consequently, exploring the utilization of audio datasets presents a promising alternative in the quest for more accessible and efficient diagnostic solutions.

**Figure 1:**
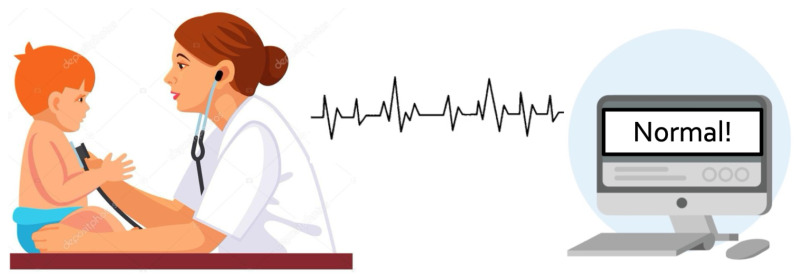
Concept of an AI-based assistance system that classifies whether heart condition is normal or abnormal using heart audio-type data.

A stethoscope is a fundamental tool for assessing the cardiopulmonary health of neonates by detecting heart sounds. Figure 1 shows a concept of an AI-based assistance system that classifies whether a heart condition is normal or abnormal using heart audio-type data. Suppose it is possible to distinguish between normal and abnormal heart disease from a stethoscope that is always used for regular health checks in infants. In that case, heart disease can be helpful for early detection. ECG or PCG is a test performed when a detailed examination is necessary at the request of the medical doctor in charge, so it is possible when the disease has worsened. Clinicians leverage this auditory data to diagnose various cardiac abnormalities. However, relying on the practitioner’s clinical acumen and experience can lead to potential oversights in early pathology detection, particularly in cases exhibiting subtle clinical manifestations.

This study aimed to develop an AI-driven support system that utilizes heart sound data to enhance diagnostic accuracy and expedite patient management in hospital settings. Electrocardiography (ECG) and Phonocardiography (PCG) are the most reliable modalities for diagnosing cardiovascular disorders. It is crucial to emphasize that our proposed methodology and findings are optimized for early detection, as the bulk of existing AI research in this domain predominantly utilizes ECG and PCG datasets for predictive modelling.

## 2. Related Works

Table 1 shows the previously proposed models on heart sound research. Electrocardiogram (ECG) and Phonocardiogram (PCG)-based studies have been used in clinical settings for decades as reliable medical technologies for diagnosing heart diseases. ECG records the heart’s electrical activity, making it practical for detecting arrhythmias, myocardial infarctions, and other electrical abnormalities [5], whereas PCG is useful for identifying physical abnormalities such as heart murmurs [6,7]. Recent research has focused on leveraging deep learning and AI techniques to automate ECG and PCG data analysis and develop models for disease prediction.

Xiao developed an AI system combining CNN and LSTM to enable early detection of congenital heart disease (CHD) in children [1]. This study integrated IoT technology to facilitate remote analysis of heart sounds, demonstrating the feasibility of incorporating smart medical devices. Islam et al. developed an electronic stethoscope and recorded children heart sound. They proposed a SVM (Support Vector Machine) model to classify the data [2,7]. They did not describe the model architecture, the number of parameters for the model, the training costs, etc. The SVM approach has many limitations; therefore, we feel better models should be proposed. Tao, Zihan developed a model that converts PCG signals into spectrogram images and applies Vision Transformer (ViT) and a Convolutional Recurrent Neural Network (CRNN) to detect heart disease [8]. Lu, Kailong proposed an advanced method for predicting heart diseases by integrating temporal and textual information for more precise analysis [9]. Additionally, Wang introduced a hybrid model combining CNN and Transformer in a parallel structure to analyze heart sound data, applying frequency transformation techniques to improve accuracy over existing models [10,11,12]. However, ECG-based analysis faces real-time processing challenges and requires sensor attachment, which limits its use for immediate screening.

PCG-based research provides a more cost-effective and accessible alternative compared to traditional ECG and echocardiography [13,14,15]. Recent advancements focus on integrating AI techniques to automate heart sound analysis for early disease detection. Radha, Kodali developed a CRNN (Convolutional Recurrent Neural Network) model that directly analyzes raw heart sound data, outperforming conventional MFCC- and spectrogram-based approaches [16]. Habijan, Marija proposed a CNN-GRU (Gated Recurrent Unit) model for heart sound classification, utilizing various signal processing techniques to remove noise and enhance accuracy [17,18]. Nguyen, Minh Tuan introduced a CNN-LSTM model incorporating log-mel spectrograms, achieving higher performance compared to conventional time-frequency transformation methods [19]. Emmanuel described the signal processing technique for heart sound analysis in clinical diagnosis [20]. Deep learning-based computer-aided heart sound analysis in children has been introduced by Liu and Rubin [18,21]. Pediatric heart sound segmentation without using the ECG has been introduced by Sepehri and Li [14,15,22,23]. Deep learning framework based on spectrograms for heart sound classification is proposed by Chen [12,24]. Children’s heart sounds at a distance with digital recordings has been proposed [17,25]. Many open access databases for the evaluation of heart sound, are available but children heart sound dataset is only proposed by Mendeley [8]. Heart sound segmentation approach is also proposed [19,20,21,22,23,24]. Chen proposed Log-Mel Spectrum Features [25]. Ren proposed Time and time–frequency features integrated CNN model [6].

**Table 1:** Previously proposed models for heart sound research.

Reference	Year	Dataset	Age	Data Type	Model	Accuracy [%]
Ref. [1]	2019	Pediatric	Children	Signal	CNN	96
Ref. [2]	2019	Mendelay	Children	Audio	SVM	94.2
Ref. [7]	2024	CirCor	Adult	PCG	CRNN	99.7
Ref. [8]	2023	PASCAL	Adult	PCG	RNN	90
Ref. [9]	2023	CirCor	Adult	PCG	CNN/LSTM	99
Ref. [10]	2012	Proposed	Adult	PCG	Diagnose	90
Ref. [13]	2010	-	Adult	ECG	MLP	93
Ref. [16]	2023	CirCor	Adult	PCG	CNN	91
Ref. [17]	2006	Proposed	Children	ECG	Analysis	93
Ref. [19]	2015	Proposed	Adult	PCG	KNN	93.3
Ref. [20]	2021	ECG	Adult	ECG	HMM	99
Ref. [18]	2001	Proposed	Children	Signal	ANN	100
Ref. [21]	2020	PhysioNet	Adult	ECG/PCG	HSMM	96
Ref. [23]	2024	Proposed	Adult	Audio	Pre-traind	58.0
Ref. [14]	2020	Not specified	Adult	ECG	CNN	97
Ref. [12]	2017	PhysioNet	Adult	PCG	CNN	83
Ref. [24]	2020	PhysioNet	Adult	PCG	CNN/MLP	98
Ref. [25]	2023	PhysioNet	Adult	PCG	CNN	71

However, several limitations remain in heart sound analysis. Noise and environmental factors can significantly affect accuracy, and diagnosing all heart diseases solely based on PCG signals remains a challenge. Additionally, PCG-based analysis still requires clinical interpretation, and to enhance diagnostic reliability, an automated system integrating smart stethoscopes and AI technology is essential. Such a system would enable heart disease detection without direct involvement of medical professionals, making PCG analysis more practical for real-world applications.

## 3. Methods

### 3.1. Dataset

In this study, we utilized a meticulously curated heart sound dataset from pediatric patients at Khulna Shishu Hospital and Khulna Fortis Hospital. Heart sounds were recorded with a stethoscope under the supervision of a qualified pediatric cardiologist, ensuring high medical standards. To train and evaluate the model, we partitioned the dataset into training (80%), validation (10%), and test (10%) sets following an 8:1:1 ratio. The data was randomly split while maintaining class distribution across subsets.

The recordings were averaged over six-second intervals. The decision to average heart sound recordings over six-second intervals are helpfull to ensure a sufficient representation of multiple cardiac cycles while maintaining a manageable data window for processing and analysis. A typical heart rate ranges from 60 to 100 beats per minute, meaning that a six-second interval captures approximately 6 to 10 heartbeats. This duration is generally long enough to capture consistent patterns in heart sounds. There is 60 distinct heart sounds: 30 associated with congenital and acquired cardiac anomalies (e.g., Ventricular Septal Defect, Atrial Septal Defect, Patent Ductus Arteriosus, Tetralogy of Fallot, Pulmonary Stenosis, Aortic Stenosis) and 30 categorized as normal. This normal baseline is critical for identifying deviations that may indicate cardiac disorders.

The dataset’s classification relied on phonocardiographic (PCG) analysis to differentiate normal from abnormal heart sounds. Healthy PCG signals are characterized by the clear presence of the two fundamental heart sounds, S1 and S2. Abnormal signals, however, display distinctive traits, such as systolic murmurs at the left upper sternal border or variations in timing relative to S1 and S2. These nuances in heart sound patterns are essential for understanding pediatric cardiac health.

### 3.2. Architecture

**Figure 2:**
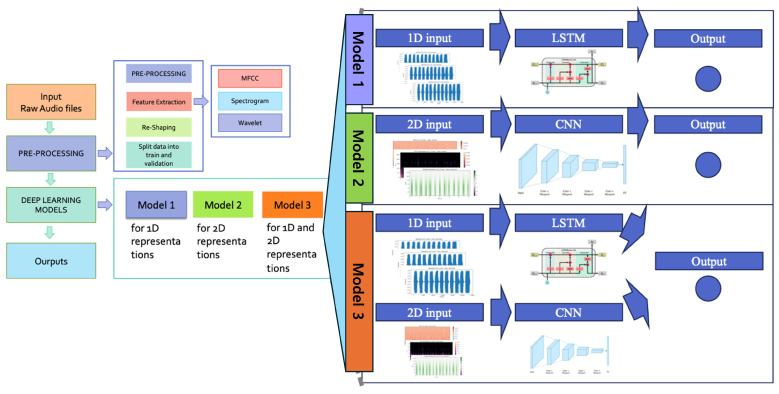
A proposed architecture for classifying cardiac conditions based on audio-type data. The left side represents the workflow, and the right represents detailed processing. We use three types of feature extractions and compare which feature extractor is suitable for the audio type. In addition, we apply three different deep learning models and compare the results to see which deep learning model is ideal for learning audio data types.

Figure 2 presents a proposed architecture for classifying heart conditions from audio-type data. The left depicts the workflow, while the right side details the processing involved. We employ three feature extraction techniques and compare their effectiveness alongside three deep learning models to determine the optimal approach for audio classification.

The first model utilizes Recurrent Neural Networks, incorporating Gated Recurrent Units (GRUs) and Long Short-Term Memory (LSTM) networks to capture temporal dependencies in raw signals. It also uses a 1D Convolutional Neural Network (CNN) for analysis. The second model transforms raw audio data into spectrograms and employs a specialized 2D CNN for classification. The final model combines 1D and 2D data streams, assessing the advantages of multimodal deep learning for improved classification.

This study contributes significantly to applying deep learning in audio signal classification, providing valuable insights for future investigations.

### 3.3. Feature Extraction

**Figure 3:**
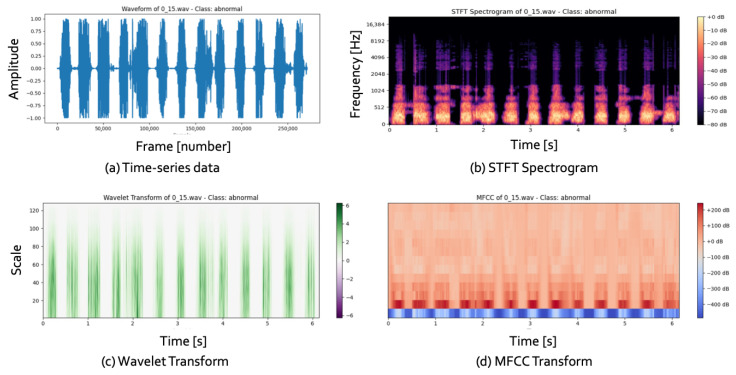
An example of 1D and 2D representations: the 1D representation in (**a**) represents time-series data, and the 2D representations in (**b**–**d**) represent the results of transformation from 1D time-series data to the image.

Figure 3 illustrates 1D and 2D representations of data, with (a) showing time-series data from audio signals and (b–d) depicting the transformed 2D images.

Cardiovascular diseases (CVDs) are a significant cause of global mortality, necessitating improved diagnostic methods. Electrocardiograms (ECGs) and heart sound analysis are essential non-invasive tools for assessing the heart’s electrical and mechanical functions. However, manual interpretation of these signals, as seen in Figure 3a, is complex and laborious, creating challenges for clinicians. Automated analysis systems offer a solution, providing faster and more accurate evaluations.

Recent studies have focused on transforming 1D time-series data into 2D representations, as shown in Figure 3b–d. Convolutional Neural Networks (CNNs) have emerged as powerful tools for image processing and have been employed for heart sound classification in CVD diagnostics. Common transformation methods include the Short-Time Fourier Transform (STFT), the Wavelet Transform, and the Mel-Frequency Cepstral Coefficient (MFCC), which result in 2D time–frequency spectrograms.

For example, Huang et al. developed a 2D CNN for classifying five arrhythmias from ECG data, achieving 99.00% accuracy compared to 90.93% for a traditional 1D CNN. Their findings suggest manual preprocessing techniques, such as signal filtering and feature selection, are unnecessary when using 2D CNNs for ECG classification.

### 3.4. Models

In our experimental framework, we implemented three distinct modelling approaches. The first utilized a Long Short-Term Memory (LSTM) architecture for anomaly detection in raw audio data, which yielded an accuracy of 66% on a dataset of children’s heart sounds. The second model was based on a Convolutional Neural Network (CNN), employing Mel-Frequency Cepstral Coefficients (MFCC), a spectrogram, and Wavelet Transforms for feature extraction. Our evaluation indicated that MFCC outperformed the other extraction techniques, as detailed in Table 2. The third approach integrated a hybrid architecture that combined CNN and LSTM, explicitly employing a 2D-CNN in conjunction with LSTM layers. This hybrid model, which we are particularly proud of, diverged from conventional architectures that mainly utilize 1D-CNNs, showcasing superior performance metrics compared to existing models in the relevant literature.

#### 3.4.1. Model 1 for 1D Representations

Our models were developed using Keras, leveraging TensorFlow as the backend. Model 1, as shown in Figure 2, employs a multi-layer LSTM architecture with three LSTM layers designed for classification tasks using three distinct window sizes. The input is raw audio, which goes through feature extraction and reshaping to a particulate shape before input.

The architecture begins with an LSTM layer comprising 128 units, followed by a dropout layer set at a rate of 0.2 to address potential overfitting. The second layer mirrors the first, featuring another 128-unit LSTM and a dropout layer with the same 0.2 rate. The third LSTM layer maintains identical specifications, containing 128 units, followed by a dropout layer at 0.2, before proceeding to fully connected layers.

We utilized the Adam optimizer for optimization, while the loss function employed during training was categorical cross-entropy. The model was trained for 50 epochs with a batch size of 32. The output layer incorporates a softmax activation function and includes two neurons, facilitating the classification of two distinct categories of children’s heart sounds.

#### 3.4.2. Model 2 for 2D Representations

The architecture of Model 2, meticulously designed for efficiency, consists of a series of convolutional layers, followed by fully connected layers optimized for processing input images of size 128 × 128 × 3. The model begins with a 2D convolutional layer that employs 32 filters, with a kernel size of 3 and a stride of 1. This is succeeded by a max pooling layer configured with a pool size of 2 and a stride of 2. Batch normalization is applied after max pooling to enhance the stability and speed of the training process.

The subsequent convolutional layer increases complexity by utilizing 64 filters, maintaining the kernel size of 3 and stride of 1. This is followed again by a max pooling layer and batch normalization. A third convolutional layer, featuring 128 filters and the same kernel and stride parameters, is introduced next, followed by another round of max-pooling and batch normalization.

After processing through the convolutional stack, the resultant feature maps are flattened and fed into fully connected layers containing 128 units. ReLU activation functions across all convolutional layers is a key design choice, introducing non-linearity into the model and ensuring its robustness. A dropout layer, with a dropout rate of 50%, follows the fully connected layers to mitigate the risk of overfitting.

The Adam optimizer, a state-of-the-art choice for training deep learning models, is utilized for the training procedure. It is paired with the categorical cross-entropy loss function to gauge performance metrics. The model is trained over 100 epochs with a batch size of 32. A softmax activation function is applied to the output layer consisting of two units, enabling classification between the two classes of pediatric heart sounds.

#### 3.4.3. Model 3 for 1D and 2D Representations

The architecture of Model 3 combines several convolutional layers and LSTM units, complemented by fully connected layers and dropout mechanisms, optimized for performance through rigorous training. It starts with a 2D convolutional layer featuring 32 filters with a 3 × 3 kernel, accepting input dimensions of (64, 64, 3) and utilizing ReLU activation. This is followed by a max pooling layer with a pool size of 2 and a stride of 2, along with batch normalization to improve stability and convergence.

The model progresses to a second convolutional layer with 64 filters, maintaining a 3 × 3 kernel and a stride of 1, again followed by another max pooling layer and batch normalization. The resulting feature maps are flattened before entering two sequential LSTM layers with 64 and 128 units, showcasing the model’s advanced architecture. A dropout layer is applied post-LSTM with a 0.2 rate to mitigate overfitting. The architecture is completed with fully connected layers.

Training is executed using the Adam optimizer, with categorical cross-entropy as the loss function. The model was trained over 50 and 100 epochs with a batch size of 32. Finally, it outputs predictions through a softmax layer with two units, showcasing its ability to accurately distinguish between normal and abnormal heart sounds in pediatric patients, demonstrating its strong diagnostic potential.

### 3.5. Model Evaluation

To evaluate the classification strategies, we utilized ten-fold cross-validation. In each fold, we computed four key performance metrics: accuracy (ACC), sensitivity (Se), specificity (Sp), and modified accuracy (MAcc). These metrics were determined using the standard definitions:

(1) $$Acc=\frac{TP+TN}{TP+FP+TN+FN}$$

(2) $$Se=\frac{TP}{TP+FN}$$

(3) $$Sp=\frac{TN}{FP+TN}$$

(4) $$MAcc=\frac{Sp+Se}{2}$$

- *TP* (true positive): the number of patients correctly identified as patients.
- *FN* (false negative): the patients incorrectly classified as healthy.
- *FP* (false positive): healthy individuals misclassified as patients.
- *TN* (true negative): healthy subjects accurately classified as healthy.

After completing the cross-validation, we calculated the average values of these parameters across the 10 folds. The entire process was repeated 50 times, with each iteration randomly selecting a set of 218 normal signals to concatenate with 218 abnormal heart sounds. This random selection process ensures that our results are free from bias. The reported average results across these 50 iterations are presented in Section 4.

## 4. Results

### 4.1. Performance Results

**Figure 4:**
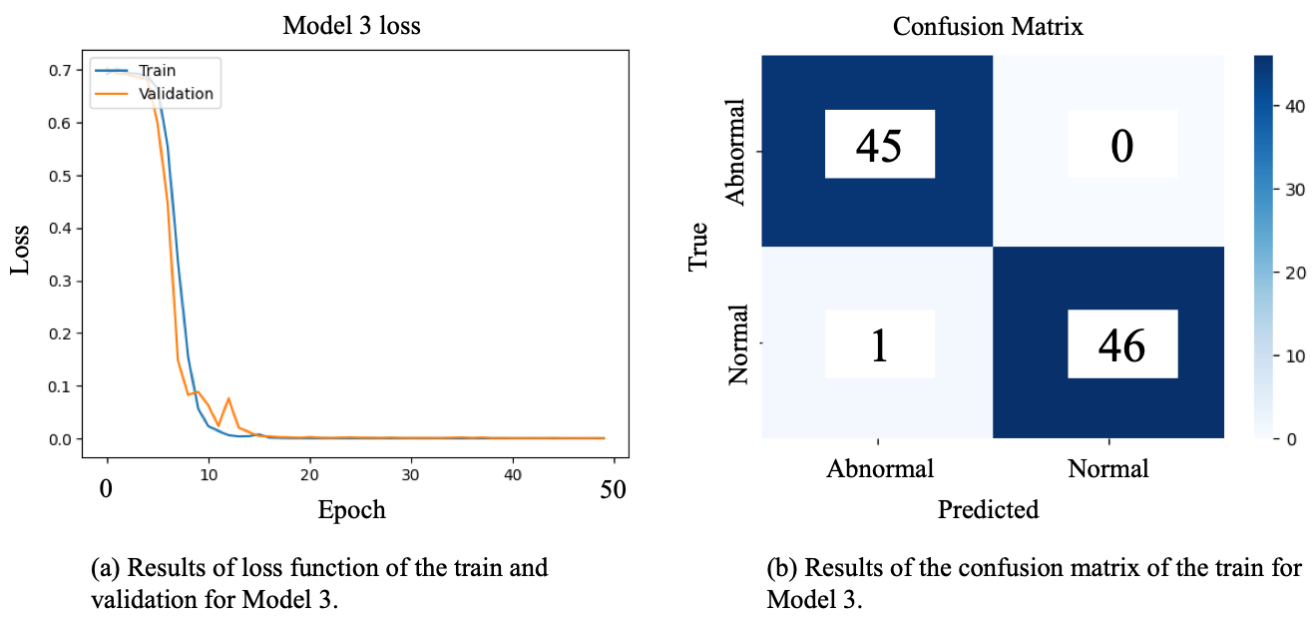
Results of training and validation for Model 3: the plot (**a**) represents the loss function, and (**b**) represents the confusion matrix.

Figure 4 illustrates the learning outcomes of Model 3. In Figure 4a, we observe the evolution of the loss metric, while Figure 4b presents the confusion matrix outcomes. Notably, the loss trajectory converges towards zero smoothly, indicating no adverse effects on learning mechanisms, such as overfitting. Performance evaluations reveal a high rate of accurate predictions across the test data, instilling confidence in the model’s performance.

Table 2 shows a comparison of our proposed model with recently introduced models. Initial analysis of the 1D representation—achieved by feeding raw time-series data directly to the model—indicates superior performance from the two LSTM models operating in tandem compared to a sole LSTM implementation. Conversely, results from the third model utilizing SVM provide promising classification outcomes. However, it warrants caution regarding potential overfitting risks inherent with nonlinear classifiers. The standalone LSTM model with Model 1 exceeds the performance of the second LSTM model but does not match the aggregate efficacy of the first two LSTMs.

Advancing to the 2D representation, where time-series data is transformed into image formats for model input, we witness a significant enhancement in overall performance compared to the raw time series method. This improvement is primarily due to the meticulous and rigorous hyperparameter tuning and variations in the preprocessing techniques. Our approach with Model 2 involved three distinct preprocessors combined with straightforward CNN architectures, leading to significant performance disparities within the same model framework, as detailed in Table 3. This underscores the critical nature of thorough hyperparameter optimization and network architecture design, where strategies to mitigate overfitting and configurations of CNN and pooling layers profoundly influence the results.

Lastly, this study delves into the effectiveness of a multimodal learning strategy that independently trains and integrates two modalities. The results for Model 3 demonstrate enhanced performance metrics, such as higher accuracy and lower loss, over benchmarks from prior research. Furthermore, Model 3 consistently surpasses Models 1 and 2, maintaining robust and reliable performance under identical experimental conditions.

### 4.2. Results Regarding Cost of Training

Table 4 offers an in-depth comparative analysis of resource expenditure associated with training each model, conducted within a consistent environment that aligns with established research methodologies. This analysis employs our proposed evaluation metrics to ensure accuracy and relevance. Prior studies have shed light on specific performance indicators, yet they also highlighted notable gaps in parameter specifications that hindered comprehensive comparisons. In our examination, Model 1, Model 2, and Model 3 were meticulously evaluated under identical conditions to facilitate a valid comparison.

Model 1, which utilizes a one-dimensional representation, consistently demonstrates lower performance across all evaluated metrics compared to its counterparts. However, it stands out for its remarkably reduced parameter count during training. This simplification correlates with significantly shorter training durations, making it highly efficient and capable of successful training, even on hardware with limited specifications.

In contrast, Model 2 employs a two-dimensional representation and delivers a balanced performance that matches contemporary studies. Despite this, the lack of prior research outlining its parameter requirements leaves us with an incomplete understanding of its scalability. It is worth noting that Model 2’s parameter count is approximately thirty times greater than that of Model 1, representing a substantial increase in resource demands, albeit in exchange for enhanced performance levels.

Model 3, utilizing a multimodal approach, achieves a slight performance boost compared to earlier research findings. Interestingly, the parameter count for Model 3 is reduced by about one-third compared to Model 2. This noteworthy reduction suggests a potential decrease in computational burden while still maintaining competitive performance capabilities, paving the way for innovative applications in the future.

These findings illuminate a crucial insight: direct modelling of time-series data does not automatically translate into superior outcomes. However, using image-converted data for training can significantly enhance performance metrics. Furthermore, the multimodal learning framework adeptly minimizes the computational costs associated with training, thereby presenting a promising direction for future developments in the field.

## 5. Discussion

A heart murmur, an auscultatory finding indicative of turbulent blood flow within the cardiovascular system, is a common occurrence in the pediatric population during outpatient evaluations [26,27,28]. It is the leading cause of referrals from primary care to pediatric cardiology clinics. The prevalence of asymptomatic heart murmurs in children is significant, ranging from 24% to 97.5%, with a peak incidence observed in the 8 to 12-year age group [29]. In newborns, the incidence is reported to be approximately 40–50 per 1000 live births, while the prevalence in school-aged children and adolescents is estimated to be 75–80%.

It is crucial to understand that only a subset of these murmurs are associated with structural heart disease; most are classified as innocuous. The responsibility of accurate differentiation between pathological murmurs, which may indicate underlying heart disease, and benign murmurs—commonly observed in healthy children—is significant during physical examination. Despite the growing reliance on echocardiography, which can diminish the necessity for auscultation, proficiency in evaluating heart murmurs remains vital. Clinicians must ascertain whether a patient should be referred to a pediatric cardiologist for further assessment or if the likelihood of significant heart disease is minimal, thus reassuring the patient and guardians.

Heart murmurs can frequently be detected in pediatric patients. While many are non-pathological, they can occasionally serve as the sole indicator of severe cardiac pathology, necessitating a careful and urgent approach. Murmurs classified as pathological typically present with specific characteristics: they may occur during systole or diastole, exhibit grade III intensity or greater, have a coarse quality, feature an abnormal second heart sound (S2) or systolic click, or be exacerbated by postural changes such as standing. In instances where a pathological murmur is suspected, immediate further evaluation with echocardiography is indicated to confirm the presence of any underlying cardiac abnormalities.

While auscultation findings are paramount, external factors such as clinic congestion, physician fatigue, and ambient noise can compromise diagnostic accuracy. Given the critical decision-making in determining the necessity for echocardiographic evaluation, the auxiliary system proposed in this study will enhance clinicians’ ability to make timely and accurate assessments.

## 6. Limitations

This study introduces an AI-driven heart disease prediction model leveraging an intelligent stethoscope. However, it is important to note that the model’s performance may be compromised due to the constrained dataset used for training. This could potentially lead to a lack of generalizability across diverse patient populations. A dataset skewed towards a specific demographic may adversely affect real-world clinical applicability. To address this, future research will involve the development of a comprehensive, large-scale dataset encompassing a wide range of age groups and pathological conditions. Importantly, we will seek multi-institutional collaborations to enhance the robustness and reliability of the model, recognizing the value of collective efforts in improving healthcare technology. In this research, our dataset was very small, which is the biggest limitation of our study. This study has several limitations that should be acknowledged. First, the dataset used was relatively small, which may limit the generalizability of the results. A larger and more diverse dataset would be more accurate in validating the findings across different populations and clinical settings. Second, the current dataset is very clean; therefore, we did not include a noise cancellation function. In real-world clinical environments, background noise can significantly affect data quality and system performance. The absence of noise handling mechanisms may therefore impact the accuracy and reliability of the system in practical applications.

Another significant limitation is the AI model’s insufficient interpretability, which challenges clinician confidence in its outputs. Deep learning algorithms’ black-box nature renders their diagnostic reasoning opaque. Consequently, forthcoming studies will employ interpretability frameworks such as Grad-CAM, SHAP, and LIME to generate visual insights into the AI system’s decision-making pathways. Collaborating with medical professionals to refine these interpretability techniques will also be a priority to foster trust among end-users.

Furthermore, the absence of comparative analyses with established diagnostic modalities like ECG and echocardiography underscores a critical gap in assessing the clinical utility of the intelligent stethoscope. To address this, future investigations will systematically compare the performance of the AI-based analytical model with traditional diagnostic approaches. Rigorous experimentation with medical practitioners will be conducted to validate the model’s efficacy in authentic clinical settings. Through these initiatives, the intelligent stethoscope aims not to replace but to complement conventional diagnostic processes, facilitating applications in remote healthcare and primary care environments.

## 7. Conclusions

In this study, we investigated the methodology for analyzing heart sound data to facilitate early detection of cardiovascular abnormalities in infants. We examined the efficacy of one-dimensional (1D) signal processing alongside two-dimensional (2D) image transformations applied to various heart sound signals, and we compared the classification performance of both image-based and signal-based deep learning (DL) models. Additionally, we assessed the validity and effectiveness of a multimodal fusion approach that integrates both 1D and 2D representations.

Our findings indicate that 1D heart sound signal networks exhibit a lower training cost than those based on 2D signal representations while outperforming the latter in classification tasks. This disparity in performance can be attributed to the 2D image transformation’s ability to encapsulate richer information, enabling simultaneous learning of temporal dynamics and frequency components. We confirmed that multimodal fusion models can effectively reduce training costs while sustaining robust performance metrics.

In conclusion, our study substantiates the viability of a multimodal approach for the early diagnosis of cardiac abnormalities in infants. This approach, which integrates both 1D and 2D representations, holds significant promise for the future of early diagnosis in this vulnerable population. It contributes valuable insights to the existing literature. It paves the way for future advancements in heart sound signal processing techniques aimed at prompting the identification of cardiac diseases in infants.

## Figures and Tables

**Table 2:** Comparison of our proposed model with recently introduced models.

Representation	Models	Dataset	Data Type	Accuracy [%]
1D representations	Ref. [7] LSTM, RNN	PASCAL	Audio	90.0
	Ref. [23] LSTM	Proposed	Audio	38.0
	Ref. [2] SVM	Mendelay	Audio	94.1
2D representations	Ref. [3] 2D Vit1D CRNN	PhysioNet	Audio	97.3
	Ref. [23] ResNet 50	Proposed	Audio	56.0
	Ref. [5] PCTMF-Net	PhysioNet	Audio	93.0
Our proposed models	Model 1 for 1D representations	Mendelay	Audio	66.7
	Model 2 for 2D representations	Mendelay	Audio	91.7
	Model 3 for 1D and 2D representations	Mendelay	Audio	98.9

**Table 3:** Performance capabilities of different feature extractors compared to the proposed model.

Feature Extractor	Precision	F1-Score	Test Accuracy [%]
MFCC	(Ab: 0.88) (N: 0.80)	(Ab: 0.82) (N: 0.84)	0.83
Wavelet	(Ab: 0.75) (N: 0.70)	(Ab: 0.71) (N: 0.74)	0.72
STFT	(Ab: 0.60) (N: 0.62)	(Ab: 0.63) (N: 0.59)	0.61

**Table 4:** Comparison of our proposed model with recently introduced models with different evolution parameters.

Models	Acc	Sp	Se	MAcc	Number of Parameters
Ref. [3] 2D Vit-1D CRNN	0.9733	0.9731	0.9735	0.9733	-
Ref. [6] TTFI-CNN	0.9715	0.9713	0.9717	0.9715	-
Our Model 1	0.6666	0.5000	0.5000	0.5000	331,010
Our Model 2	0.9167	0.8333	1.000	0.9167	9,914,309
Our Model 3	0.9891	0.9894	0.9894	0.9894	3,346,370

## Data Availability

The dataset used in this study is a public dataset that anyone can download and use for model training in (accessed on 1 March 2024) [https://data.mendeley.com/datasets/5447z7m2rr/1].

## Abbreviations

The following abbreviations are used in this manuscript:

MFCCs	Mel-Frequency Cepstral Coefficients
ECG	Electrocardiogram
PCG	Phonocardiogram
CHD	Congenital Heart Disease
LSTM	Long Short-Term Memory
CNN	Convolutional Neural Network
FFT	Fast Fourier Transform
DWT	Discrete Wavelet Transform
STFT	Short-Term Fourier Transform
CVD	Cardiovascular diseases
MAcc	Mean Accuracy
